# From data to decision: Machine learning determination of aerobic and anaerobic thresholds in athletes

**DOI:** 10.1371/journal.pone.0309427

**Published:** 2024-08-29

**Authors:** Michał Tomaszewski, Anna Lukanova-Jakubowska, Edyta Majorczyk, Łukasz Dzierżanowski

**Affiliations:** 1 Faculty of Electrical Engineering, Automatic Control and Informatics, Opole University of Technology, Opole, Poland; 2 Faculty of Physical Education and Physiotherapy, Opole University of Technology, Opole, Poland; Instituto Politecnico de Setubal, PORTUGAL

## Abstract

Lactate analysis plays an important role in sports science and training decisions for optimising performance, endurance, and overall success in sports. Two parameters are widely used for these goals: aerobic (AeT) and anaerobic (AnT) thresholds. However, determining AeT proves more challenging than AnT threshold due to both physiological intricacies and practical considerations. Thus, the aim of this study was to determine AeT and AnT thresholds using machine learning modelling (ML) and to compare ML-obtained results with the parameters’ values determined using conventional methods. ML seems to be highly useful due to its ability to handle complex, personalised data, identify nonlinear relationships, and provide accurate predictions. The 183 results of CardioPulmonary Exercise Test (CPET) accompanied by lactate and heart ratio analyses from amateur athletes were enrolled to the study and ML models using the following algorithms: Random Forest, XGBoost (Extreme Gradient Boosting), and LightGBM (Light Gradient Boosting Machine) and metrics: R^2^, mean absolute error (MAE), mean squared error (MSE) and root mean square error (RMSE). The regressors used belong to the group of ensemble learning algorithms that combine the predictions of multiple base models to improve overall performance and counteract overfitting to training data. Based on evaluation metrics, the following models give the best predictions: for AeT: Random Forest has an R^2^ value of 0.645, MAE of 4.630, MSE of 44.450, RMSE of 6.667; and for AnT: LightGBM has an R^2^ of 0.803, the highest among the models, MAE of 3.439, the lowest among the models, MSE of 20.953, and RMSE of 4.577. Outlined research experiments, a comprehensive review of existing literature in the field, and obtained results suggest that ML models can be trained to make personalised predictions based on an individual athlete’s unique physiological response to exercise. Athletes exhibit significant variation in their AeT and AT, and ML can capture these individual differences, allowing for tailored training recommendations and performance optimization.

## Introduction

Lactate analysis plays an important role in sports science [1, 2] and an exploration of lactate’s function in both anaerobic and aerobic glucose metabolism, as well as its connection to various exercise intensities, has spanned over two centuries, giving rise to ongoing debates. The introduction of concepts such as lactate turn point, lactate threshold, onset of blood lactate accumulation (OBLA), maximal lactate steady state (MLSS), anaerobic threshold, ventilatory threshold, lactate equivalent, lactate minimum speed, and individual anaerobic threshold has resulted in confusion and challenges in their widespread application for performance evaluation [3]. Derived from the examination of energy provision and lactic acid generation in progressive exercise, a three-phase model can be established, delineating two distinct thresholds: aerobic (AeT) and anaerobic (AnT) [4]. Thus, both thresholds significantly determine an athlete’s performance and endurance [1, 2].

AeT and AnT are critical markers for an athlete’s fitness and performance levels in performance assessment. They provide insights into an athlete’s physiological capacity and can be used to track improvements or declines over time. These thresholds signify the body’s ability to manage and clear lactic acid, a byproduct of intense exercise that can cause fatigue. A higher AeT and AnT reflect a more efficient and capable physiological system, and they are directly associated with better endurance [1]. Athletes with elevated thresholds can sustain high-intensity exercise for longer durations without experiencing significant fatigue or performance deterioration. This enhanced endurance is a critical factor in sports that demand prolonged periods of exertion, such as endurance running, cycling, or triathlons [5–7].

AeT and AnT are associated with different energy systems in the body. Knowing these thresholds helps athletes target the right energy system during various types of sports, whether it’s aerobic (below AeT) or anaerobic (above AnT). Training within the AeT and AnT zones aligns exercise intensity with the athlete’s physiological capacities. This targeted approach leads to more effective and efficient training [1]. Athletes can avoid the pitfalls of training too intensely, which can lead to premature fatigue and overtraining, burnout with potential severe physical and psychological consequences, and injury, or training too lightly, which may not yield the desired performance improvements.

Moreover, understanding the AeT and AnT of individual players in team sports can influence tactical decisions [8, 9]. For example, a coach might use this information to determine when to make substitutions or tactical changes based on an athlete’s current energy levels. Therefore, athletes and coaches use AeT and AnT for precise determination of the most effective training intensities and to fine-tune their training programs and prevent overtraining.

Overtraining is a common concern in the world of sports, where athletes continually push their limits to achieve peak performance. When AeT and AnT data reveal signs of overtraining, athletes and coaches can take immediate action [10]. This might involve reducing training intensity or volume, incorporating more rest days, or modifying the training program to allow for recovery [11]. These adjustments help prevent excessive physical and mental stress, ensuring that the athlete remains on a sustainable and progressive training trajectory. Moreover, overtraining can lead to an increased risk of injury due to the cumulative stress placed on the body. By heeding the signs provided by AeT and AT, athletes and coaches can mitigate this risk. A well-balanced training plan that considers these thresholds not only prevents overtraining but also reduces the likelihood of overuse injuries, muscle imbalances, and other training-related health issues.

In summary, it is considered that research on AeT and AnT contributes to the development of training programs and performance enhancement strategies, and despite many controversies amongst sports scientists [12], they play a significant role in determining an athlete’s performance and endurance. Furthermore, innovations in sports science may enable more precise measurements and predictions of these thresholds. One could propose that within the realm of technologies bolstering sports science, machine learning (ML) stands out as a significant contributor [13].

ML plays an increasingly important role in predictive tasks in many fields [14]. For sport science, various intelligent algorithms can predict AeT and AnT due to their ability to handle complex, high-dimensional data and make accurate predictions [15]. ML models are data-driven, which means they can analyze large datasets that include a variety of physiological and performance metrics. AeT and AnT are influenced by numerous factors, such as heart rate (HR), oxygen consumption and blood lactate levels [15–17]. ML can effectively analyze these multidimensional data sets to identify patterns and relationships that might be challenging for traditional statistical methods. ML models can be trained to make personalized predictions based on an individual athlete’s unique physiological response to exercise. Athletes exhibit significant variation in their AeT and AnT, and ML can capture these individual differences, allowing for tailored training recommendations and performance optimization [15, 16]. Moreover, ML models can integrate data from various sources, such as training logs, biometric sensors, weather conditions, and more.

This holistic approach allows for a comprehensive assessment of factors affecting AeT and AnT, improving the quality of predictions. Parallelly, ML models have the potential to provide more accurate predictions of AeT and AnT compared to traditional methods [15–17]. These models can be fine-tuned and trained on extensive datasets, continuously improving their prediction accuracy over time. It is suggested that ML can identify the most relevant features or variables that contribute to the prediction of AeT and AnT. This feature selection process can help researchers and coaches focus on the most critical factors that impact an athlete’s thresholds, streamlining data collection and analysis. The application of ML to AeT and AnT research can lead to new insights and discoveries. These models can uncover previously unrecognized relationships and factors influencing an athlete’s physiological thresholds, contributing to the advancement of sports science, including precise and optimal determination of both anaerobic and aerobic thresholds [15, 16].

The aim of the study was to determine AeT and AnT thresholds using ML modelling and to compare the obtained results with the values determined using conventional methods. Based on the above data on AeT and AnT thresholds and ML, the research objectives and the underlying structure of the article are built upon the following fundamental components:

- investigation of AeT, AnT determination methods in related works. The first core element involves a comprehensive exploration of various techniques employed to determine both thresholds. This entails a detailed examination of the methodologies and approaches used in the field;
- experimental determination of AeT and AnT. The next critical step is to perform performance tests on a group of athletes and collect measurement material. On this basis, in accordance with the observations and modifications presented in the literature review, AeT and AnT thresholds were determined in a controlled and systematic way;
- ML models development. A significant facet of the research entails the creation and refinement of a ML model. This model is designed to leverage the data acquired through the experiment to enhance the precision and predictive accuracy of AeT and AnT threshold determination. ML algorithms are harnessed to extract valuable patterns and insights from the data, contributing to a more robust understanding of these thresholds;
- communication of research findings. The ultimate phase centers on the presentation of the research outcomes. The findings, interpretations, and implications drawn from the study are elucidated and communicated to the scientific community. This step serves to disseminate valuable knowledge and insights, advancing the collective understanding of AeT and AnT determination methods, thereby contributing to the broader field of sports science and athlete performance optimization.

## Literature review

To establish parameters for endurance training, for both professional and amateur athletes, several systems are used. Thus, the determination of heart rate zones is a crucial element which can be based on the traditional approach, referring to the level of exercise intensity, as presented by the American College of Sports Medicine [18] or to the maximum heart rate, heart rate reserve or oxygen uptake reserve [19–21]. This approach was a great help for athletes, both amateurs and professionals, as well as their coaches in planning the training program. However, it has many drawbacks, such as the difficulty in determining the maximum heart rate, mainly due to the impression of the approach of relating it to the athlete’s age. The standard deviation of this simple and popular method is reported to be ± 10–12 bpm and it consistently overestimates maximal HR in younger adults and underestimates maximal HR in older adults [20]. Nevertheless, there is strong evidence [5–7] showing that determination of exercise intensities as percentages of VO_2_max or HRmax is inadequate due to other reasons as well: individual variability, age-related decline, fitness level and lack of precision. Studies show that the assessment of training intensity is much more accurate when based on identifying target zones based on metabolic variables [22]. As exercise intensity rises, the human body adapts its energy utilization mechanisms based on immediate demands. Three distinct phases of energy utilization, marked by diverse metabolic processes, are identified and delineated by two threshold points known as AeT and AnT [1, 2, 12, 13, 23, 24]. The mentioned thresholds can be determined during a progressive exercise test by monitoring heart rate (runners) or power output (cyclists) and measuring either blood lactate concentration or the ventilation and gas exchange (V0_2_ and VC0_2_) indices [23–25]. Visually the AeT can be indicated as the point on the lactate curve where it clearly deviates from the resting baseline and the AnT as a point of accelerated lactate accumulation. Anoula and Rusko [26] define these points approximately 2 mmol x 1–1 and 4 mmol x 1–1, but they also indicate that it is necessary to take individual levels of lactate concentration into account. The most popular analytical method for determining AnT based on lactate concentration is D-max, which involves connecting a straight line between the first and last measurement points during a graded maximal exercise test and determining the point on the lactate curve furthest from this line [20, 25, 27]. Another approach is a respiratory gas exchange analysis. It allows for the application of automatic threshold detection, which in turn not only speeds up result acquisition but also enhances the precision of their detection [28]. One of the most commonly used and regarded as optimum [29] is the ventilatory equivalent method. It enables determining both anaerobic and aerobic thresholds, often described as Ventilatory Threshold 1 (VT1) and Ventilatory Threshold 2 (VT2). Ventilatory equivalent refers to the ratio of ventilation (the volume of air breathed per minute, denoted as VE) to the volume of oxygen consumed (VO_2_) or carbon dioxide produced (VCO_2_). During incremental exercise, as intensity increases, there comes a point where the ventilatory equivalent for oxygen (VE/VO_2_) or the ventilatory equivalent for carbon dioxide (VE/VCO_2_) exhibits a noticeable increase. This increase is associated with the transition from aerobic to anaerobic metabolism. The point AnT which this increase occurs is often used to determine the ventilatory threshold, which can be an indicator of the onset of anaerobic metabolism [26, 28, 30].

Determining AeT and AnT thresholds presents several challenges: from the lack of consensus among scientists on the precise definition of these concepts [sources], to the drawbacks of traditional laboratory methods (e.g., drawing blood from athletes during intense exercise, which is challenging in itself but shows lactate concentration at specific intervals). Establishing these thresholds for later use in creating training plans required the involvement of an experienced expert who could accurately interpret the collected data. There is a growing belief that other easily measurable factors, such as HRV can help automatically determine cardiorespiratory fitness measures using ML, as supported by current scientific research [31].

Recently there has been a growing emphasis that different thresholds derived from heart rate variability (HRV) using various indices could serve as potential alternatives. However, according to [32, 33] there is still a lack of a systematic review assessing how closely these HRV-derived thresholds align with established threshold concepts.

## Materials and methods

### Performance test

The described analysis exclusively employed anonymized data collected independently of this study. Performance tests were conducted on a broad group of athletes (183 people) involved in amateur sports. The respondents are men (147) and women (36) aged 16–62 (mean: 36.1, std: 10.84 yrs). These are athletes who are amateur runners and take part in running marathons (asphalt and trail). Those surveyed train on average 3–4 times a week, usually running training sessions. Determination of capacity was conducted using the graded-to-failure test, CardioPulmonary Exercise Test (CPET), performed on an electric treadmill, is one of the most effective tests for assessing the level of aerobic capacity and provides excellent opportunities to model running training based on test parameters such as VO_2_ max, VE or lactic acid level AnT individual stages of load (speed on the treadmill). The subjects performed the graded-to-refuse test once. The aim of the study was to determine the body’s training level, assess its physiological state and determine five heart rate energy zones:

- Zone 1—heart rate at recovery level,
- Zone 2—heart rate at the level of aerobic endurance development,
- Zone 3—heart rate at the level of aerobic power shaping (mixed zone),
- Zone 4—heart rate at the level of the aerobic-anaerobic threshold,
- Zone 5—heart rate in the anaerobic endurance zone.

To designate individual zones, it is necessary to determine the AeT and AnT thresholds, because the AeT threshold is the final boundary of heart rate zone 2, while the AnT threshold is the final boundary of zone 4.

Before the study, the subjects were informed about the purpose and use of the test, the parameters that would be recorded and the possibility of resigning from the test at any time, giving reasons. They also gave written informed consent to participate in the study.

Before starting the study, the athletes were medically qualified to perform the exercise. Each of the subjects was recommended to perform an initial 12-lead electrocardiogram (ECG) which was conducted using an EKG device—(BTL 08MT EKG no. 1230, BTL Industries Limited, Stevenage, Hertfordshire, UK).

The exercise test was preceded by a 10-minute individual warm-up. The test consisted of three 3-minute loading stages (3 minutes—6 km/h, 3 minutes—8 km/h, 3 minutes—10 km/h), and then the treadmill speed increased by 2 km/h every 2 minutes until 22 km/h not separated by a break, with each subsequent stage performed at a higher treadmill speed. During the test, respiratory indicators such as minute ventilation VE l/min and oxygen uptake VO_2_max (l/min/kg) were recorded. The test was carried out on a Cosmed electric treadmill (Cosmed Srl, Italy) with a speed range of up to 24 km/h. In the last 15 seconds of each 3-minute exercise and then 2-minute exercise, arterialized blood was collected from the fingertip to determine the lactate concentration. Individual measurements were recorded in the load ranges of 8–20 km/h for lactic acid level and were marked respectively: la_8, la_10, la_12, la_14, la_16, la_18, la_20. Lactate concentration was determined using the Super GL2 analyzer by Dr. Mueller (Dr. Müller Gerätebau GmbH, Freitel, Germany). The Super GL instruments family uses an electrochemical method using enzymatically sensitized biosensors for lactate detection. The measurement error of the method is less than 1.5%. During the entire test, the heart rate was recorded in order to determine the moment of energy changes illustrated by the lactic acid curve and the maximum heart rate (hr_max) recorded during the test in the load ranges of 6–22 km/h and were marked respectively: hr_6, hr_8, hr_10, hr_12, hr_14, hr_16, hr_18, hr_20, hr_22. Heart rate was recorded using the Polar system (Polar Electro Oy, Kempele, Finland).

### Description of measured and calculated parameters

Maximal Oxygen Consumption, also called Maximal Oxygen Uptake or Maximal Aerobic Capacity (VO_2_max), represents the maximum amount of oxygen an organism can absorb per unit of time (per minute), typically expressed in l/min or ml/min/kg. It is a crucial indicator of physical fitness, especially aerobic endurance in disciplines emphasizing endurance. This includes activities where both aerobic and anaerobic processes contribute, lasting from 30 seconds to 3 minutes. A higher VO_2_max signifies greater capacity for sustained intense work without fatigue symptoms. Low VO_2_max triggers additional anaerobic energy sources during exertion, leading to the production of lactic acid as a byproduct, causing localized and eventually systemic fatigue due to acidification. A higher VO_2_max allows the body to supply more oxygen to working muscles, enabling anaerobic processes at higher exercise intensities. Record values for men and women are 95 ml/kg/min and 80 ml/kg/min, respectively (e.g., cross-country skiers, cyclists). Maximum oxygen uptake depends on factors such as blood properties, oxygen transport capacity (red blood cell count, hemoglobin levels), cardiac output, respiratory system efficiency, and muscle oxidative capacity.

Ventilatory Equivalent (VE) refers to the ratio of ventilation—the volume of air breathed per minute.

Restitution/Recovery (R) is the time measured after exertion in a graded exercise test. It is determined by the post-exercise heart rate drop to 60% of the maximum heart rate achieved during the test. A recovery time within 3 minutes is considered good, while a prolonged recovery after three minutes indicates fatigue or irregularities in the training process related to excessive intensity or volume of exercise.

Maximal Heart Rate (HRmax) is the maximum heart rate during exercise, frequently occurring during high-intensity efforts or competitions. In a graded exercise test on a treadmill, reaching the true maximum heart rate is challenging due to the gradual nature of the exertion and the maintenance of acid-base balance at each stage. Psychological factors, such as fear of falling off the treadmill, often prevent participants from reaching their true maximum potential.

Respiratory Factor (RF) indicates how much of the oxygen extracted from the lungs into the bloodstream (VO_2_) is utilized in cellular oxidative processes. It provides insight into the efficiency of oxygen utilization at the cellular level.

The lactate curve, also known as the blood lactate curve, is a graphical representation of the relationship between exercise intensity and the concentration of lactate in the blood during incremental exercise. It is a fundamental tool used in exercise physiology to understand how the body responds to increasing levels of physical effort.

The values calculated and determined based on trained ML models values were:

- AeT—aerobic threshold, the parameter was determined using an expert visual method where the AeT can be indicated as the point on the lactate curve which clearly deviates from the resting baseline. It based on the physiological effect that the aerobic system is fully activated after 3 minutes of exercise and consequently, the lactic acid is removed from the blood and is converted to glucose/glycogen. As a result, the lowest level of lactate in blood is usually recorded in the last minute of the third stage of CPET test at 10 km/h. These findings are based on a practice observation. In contrast to theoretical models, a practice shows that the lowest level was detected not only at the beginning of the test, but decreases during the test when the load increases: at the beginning of exercise lactic acid is at the level of 4–5 mmol/l, and at 10 km/h it drops to about 1.8 mmol/l, but also cannot to decrease below 2 mmol/l. In such a case, AeT was determined at the level that was recorded on the last minute of the preceding load, where the lowest point of the lactate curve was observed. Due to this, in the practice approach, ensuring a safe point that prevents from entering the mixed zone while aerobic training was performed.
- AnT—anaerobic threshold, the parameter was determined using a standard D-Max method [20, 25, 27].

### The dataset and data collection process and ML modelling

**Fig 1:**
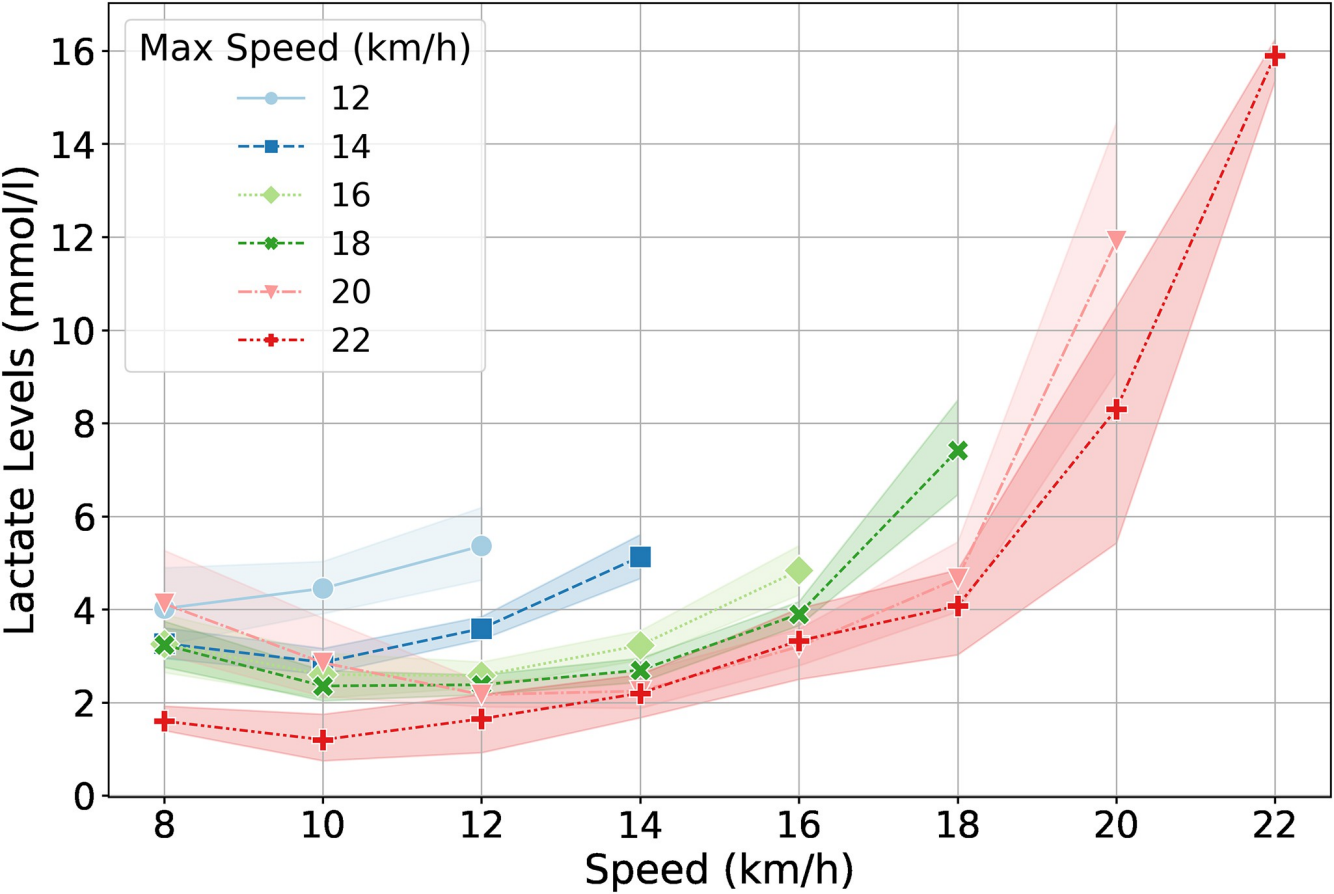
Lactic acid curves recorded during performance tests for all observations.

During a research experiment, many performance tests for different athletes were performed. After preprocessing (removing missing data and outliers) resulted in 183 complete measurement reports. Each examination was preceded by an interview with the athlete, during which additional information was obtained. Based on the collected and measured data, a dataset was built containing the following predictive features: ‘age’, ‘height’, ‘weight’, ‘VO_2_max’, ‘VE’, ‘R’, ‘HRmax’, ‘RF’, ‘VO_2_max_l_m’. The dataset was supplemented with features representing information obtained from lactic acid curve measurements. Lactic acid curves recorded during individual performance tests are shown in Fig 1.

As can be seen in Fig 1, it was not possible to obtain a curve in the full load range (6–22 km/h) in every case, which was due to the level of training and capabilities of individual athletes. For this reason, it was decided to add features to the dataset representing the level of lactic acid (features: la_8’, ‘la_10’) and heart rate (features: ‘hr_6’, ‘hr_8’, hr_10’) observed for a load of 6, 8 and 10 km, respectively. Additionally, features representing characteristic points occurring on the lactic acid curve in each of the observed cases were introduced, respectively:

- ‘la_min’—the lowest observed value of the lactic acid level,
- ‘hr_min_la’—heart rate value corresponding to the lowest observed value of the lactic acid level,
- ‘la_max’—the highest observed value of the lactic acid level,
- ‘hr_max_la’—heart rate value corresponding to the highest observed value of lactic acid level,
- ‘la_max_speed’—load at which the highest value of lactic acid level was observed.

The specific characteristics and conditions under which the data was gathered, including individual athlete variability and incomplete load ranges, underscore the importance of contextualizing the findings and highlighting the models’ potential adaptability and applicability in diverse athletic training environments.

The specific conditions under which the data was gathered, such as the level of athlete’s training, their physiological responses, and the load range capabilities, could influence the model’s applicability in real-world scenarios. For instance, athletes with higher training levels might exhibit different lactic acid curve patterns compared to less-trained individuals, affecting the model’s predictions. Additionally, the real-world application of these models may require calibration based on individual athlete profiles and conditions, such as their training stage, hydration status, nutrition, and time of day.

### The ML algorithms, metrics, and feature importance analysis

During the research, three ML algorithms belonging to the ensemble learning group were used: Random Forest, XGBoost (Extreme Gradient Boosting), and LightGBM (Light Gradient Boosting Machine). Ensemble learning algorithms are ML techniques that combine the predictions of multiple base models (often called “weak learners”) to improve overall predictive performance.

Random Forest (RF) [34] is a versatile ensemble learning method that combines multiple decision trees. It works by creating a collection of decision trees during the training phase, each of which is built using a subset of the data and a random selection of features. The final prediction is made by aggregating the individual tree predictions, typically through majority voting for classification problems or averaging for regression tasks.

XGBoost (XGB, Extreme Gradient Boosting) [35] is a gradient boosting framework known for its efficiency and performance. It builds a strong predictive model by iteratively adding decision trees to minimize the prediction errors of the previous trees. It employs gradient boosting, which means that each new tree corrects the errors made by the ensemble of the previous ones. XGBoost uses a regularized objective function to prevent overfitting and is highly customizable, allowing for fine-tuning of parameters.

LightGBM (LGBM, Light Gradient Boosting Machine [36] is a gradient boosting framework developed by Microsoft. It’s known for its speed and efficiency in handling large datasets in many fields (f.e.: [37, 38]) LightGBM uses a histogram-based approach to build decision trees, which reduces memory usage and speeds up training. It also employs a leaf-wise tree growth strategy to optimize the splits at each level, resulting in faster convergence and better accuracy. These ensemble learning algorithms have proven to be highly effective in improving predictive performance and are commonly used by data scientists and ML practitioners for a wide range of tasks. The choice of which algorithm to use often depends on the specific problem, the dataset size, and the desired trade-off between model complexity and predictive accuracy.

In turn, R^2^, mean absolute error (MAE), mean squared error (MSE) and root mean square error (RMSE) metrics were used for evaluating the performance of regression models. R^2^, also known as the coefficient of determination, is a statistical measure that represents the proportion of the variance in the dependent variable that is explained by the independent variables in a regression model.

MAE, also known as MEA, measures the average absolute differences between the predicted values and the actual values. It provides a measure of the magnitude of errors without considering their direction (overestimation or underestimation). MAE is calculated as the average of the absolute differences between the predicted values and the true values for each data point:

$$\begin{array}{c} \text{MAE}=\frac{1}{n}\sum_{i=1}^{n}\left|{\hat{y}}_{i}-y_{i}\right| \end{array}$$ (1)

MSE quantifies the average squared difference between the predicted values and the actual values in a dataset. MSE is calculated using the following formula:

$$\begin{array}{c} \text{MSE}=\frac{1}{n}\sum_{i=1}^{n}{\left(y_{i}-{\hat{y}}_{i}\right)}^{2} \end{array}$$ (2)

RMSE measures the square root of the average of the squared differences between predicted values and actual values. It penalizes larger errors more than smaller ones because it squares the differences. RMSE is sensitive to outliers in the data. RMSE is calculated as the square root of the average of the squared differences between the predicted values and the true values for each data point:

$$\begin{array}{c} \text{RMSE}=\sqrt{\frac{1}{n}\sum_{i=1}^{n}{\left({\hat{y}}_{i}-y_{i}\right)}^{2}} \end{array}$$ (3)

To analyse the feature importance of the built dataset, the SHAP tool was used. SHAP (SHapley Additive exPlanations) [39] is a framework and a set of techniques used in ML to explain the predictions made by complex models. SHAP values provide a way to understand the contribution of each feature to a particular prediction. The concept of SHAP values is based on cooperative game theory and the Shapley value, which is a way to distribute a value among a group of contributors fairly.

SHAP is model-agnostic, meaning it can be applied to many ML models, including decision trees, random forests, neural networks, and more. SHAP values provide an interpretation of feature importance for a specific prediction. They tell how much each feature contributed to the model’s prediction for a given data point. SHAP values can be visualized in various ways, making it easier to understand the explanations. Common visualizations include summary plots, force plots, and individual feature importance plots. By using SHAP, data scientists can gain insights into model behaviour and build trust in their ML models. It has become a valuable tool in the field of interpretability and explainable AI.

To prevent overfitting in the ML models, several widely recognized techniques were used. The dataset was split into training (80%) and testing (20%) sets, and regularization methods inherent to Random Forest, XGBoost, and LightGBM were incorporated. These algorithms utilize mechanisms like bagging, feature randomness, and regularization parameters to control model complexity. Additionally, feature engineering was employed, and model performance was evaluated using multiple metrics (R^2^, MAE, MSE, RMSE) to ensure robust and generalizable results.

## Results

**Fig 2:**
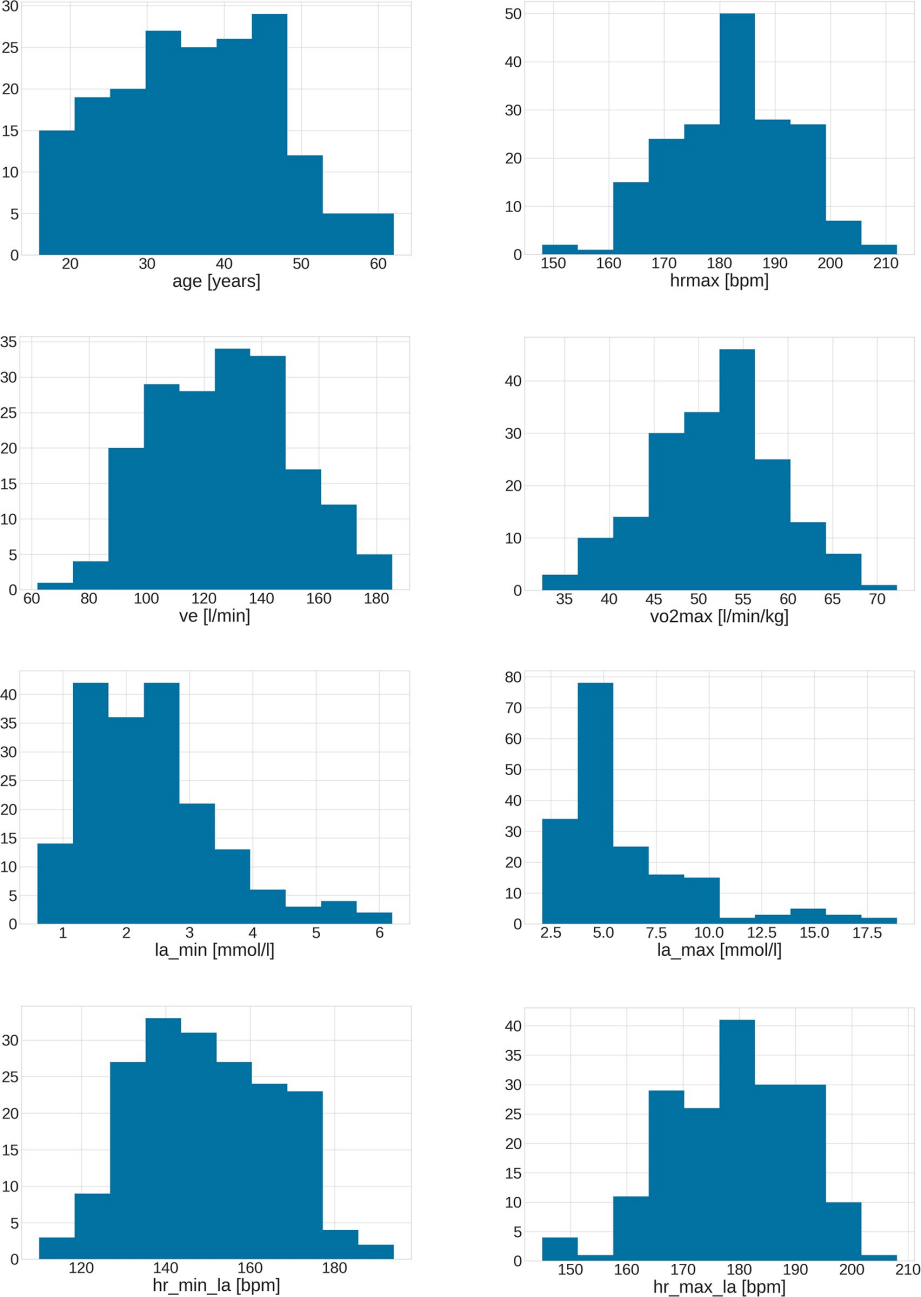
Empirical distribution of selected features of the dataset.

Fig 2 illustrates the empirical distribution of selected features within the constructed dataset, depicted in the form of histograms. The age distribution spans from 16 to 62 years, with the majority of individuals falling within the middle age range of 30 to 50 years. This demographic distribution is reflective of the diverse age groups encompassed in the dataset. The dataset captures a wide range of maximum heart rates, spanning from 148 to 212 bpm (beats per minute). Notably, a substantial number of individuals exhibit maximum heart rates falling within the range of 180 to 190 bpm, highlighting a significant concentration of data points in this specific heart rate zone.

Ventilation rates observed in the dataset range from 62 to 185 liters per minute (l/min). These values represent the diverse breathing patterns and respiratory responses among the subjects, offering insights into the variability within the dataset. The dataset encompasses VO_2_max values spanning from 32 to 72 milliliters per minute per kilogram (ml/min/kg). The most prominent cluster within the dataset corresponds to individuals with an average training level, reflecting VO_2_max values falling within the range of 45 to 60 ml/min/kg.

Additionally, the figure provides a visual representation of features related to the lactic acid curve, specifically ‘la_min’, ‘la_max’, ‘hr_min’, and ‘hr_max’. These features offer valuable insights into the lactic acid response and heart rate characteristics within the dataset, shedding light on the physiological responses of the study participants during exercise.

**Fig 3:**
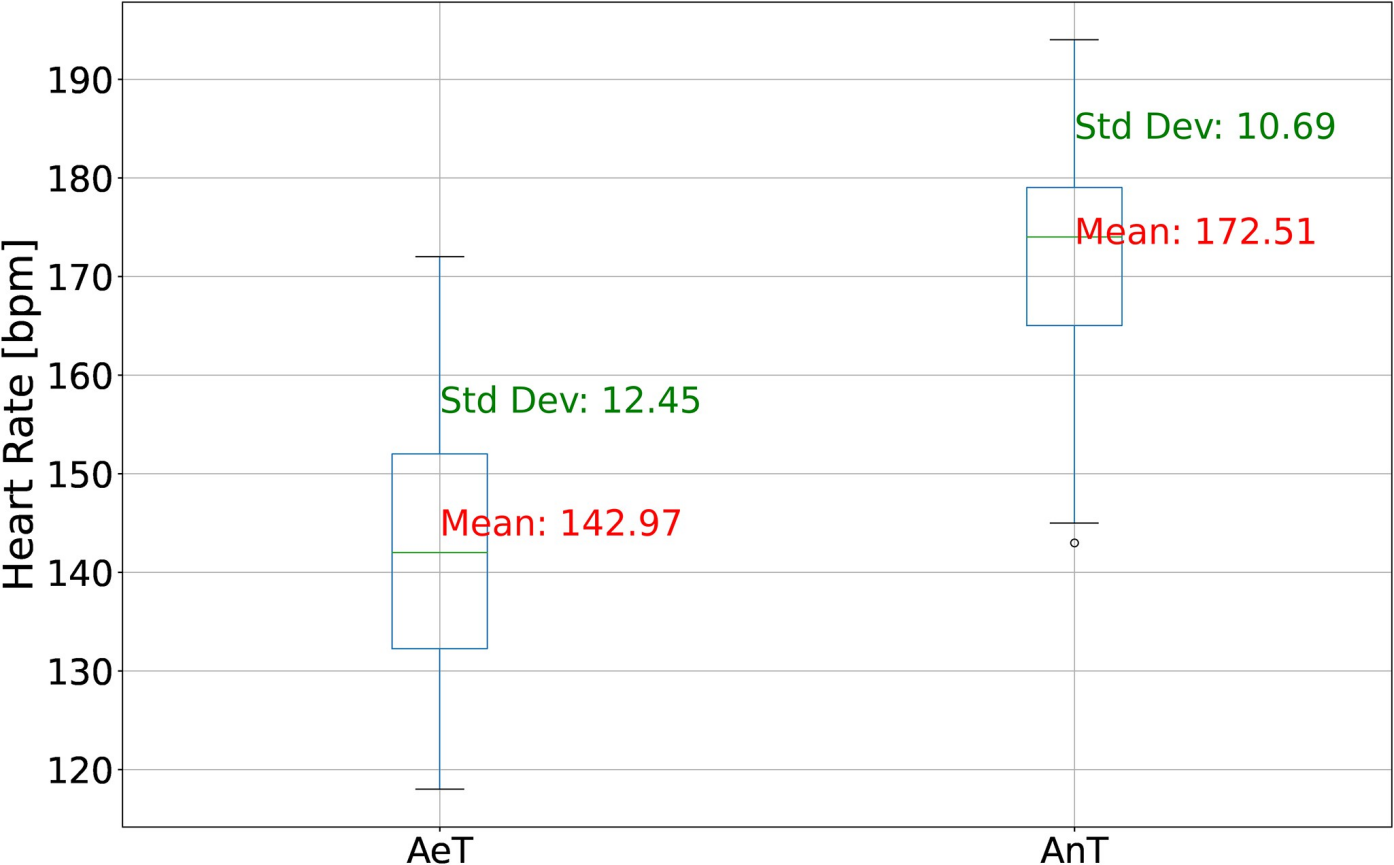
Descriptive statistics of AeT and AnT.

After each performance test, AeT and AnT thresholds were determined for each observation. Descriptive statistics of discussed thresholds are presented in Fig 3.

The mean AeT is approximately 142.66 bpm, and the mean AnT is about 172.32 bpm, serving as central indicators of the typical AeT and AnT levels within the dataset. These values provide a sense of the average thresholds observed. In terms of variability, the standard deviation for AeT is approximately 12.29 bpm, while for AnT, it’s about 10.66 bpm. These standard deviations signify the degree of variation or spread in the data. A higher standard deviation implies that the data points are more dispersed, potentially indicating relatively high variability in AeT and AnT levels among the athletes in the sample. Examining the data distribution, the range of AeT recorded is 118–172 bpm, and the range of AnT is 143–194 bpm. These values represent the minimum and maximum observed AeT and AnT levels within the dataset. The median (50th percentile) of thresholds is respectively 142 bpm for AeT, and 174 bpm for AnT.

The described dataset was used to build and evaluate various ML models aimed predicting AeT and AnT thresholds. During the modeling, the best results were achieved for regressors based on decision trees. The results are presented in Table 1.

**Table 1:** AeT and AnT threshold prediction results using different ML algorithms.

Threshold	Algorithm	R^2^_train_	R^2^	MAE	MSE	RMSE	MAPE%
AeT	RF	0.956	0.645	4.630	44.450	6.667	3.238
	XGB	0.986	0.674	5.007	40.746	6.383	3.545
	LGBM	0.965	0.599	5.587	50.133	7.080	3.913
AnT	RF	0.970	0.789	3.863	22.523	4.746	2.213
	XGB	0.995	0.716	4.285	30.253	5.500	2.459
	LGBM	0.965	0.803	3.439	20.953	4.577	1.978

The following results were obtained for AeT prediction in particular algorithms:—Random Forest with R^2^ value of 0.645, which indicates that approximately 64.5% of the variance in AeT is explained by the model. The Mean Absolute Error is 4.630, the Mean Squared Error is 44.450, and the Root Mean Squared Error is 6.667.—XGBoost with R^2^ of 0.674, MAE of 5.007, MSE of 40.746, and RMSE of 6.383. This model seems to have slightly better R^2^ and slightly worse MAE compared to the Random Forest model.—LightGBM with an R^2^ of 0.599, MAE of 5.587, MSE of 50.133, and RMSE of 7.080. This model has the lowest R^2^ and the highest MAE and RMSE among the three models, suggesting it may not perform as well in predicting AeT.

When predicting the AnT value, better results were obtained:—Random Forest has an R^2^ of 0.789, indicating that approximately 78.9% of the variance in AnT is explained by the model. The MAE is 3.863, MSE is 22.523, and RMSE is 4.746.—XGBoost has an R^2^ of 0.716, MAE of 4.285, MSE of 30.253, and RMSE of 5.500. This model appears to have a lower R^2^ and higher MAE compared to the Random Forest model.—LightGBM has an R^2^ of 0.803, the highest among the models, MAE of 3.439, the lowest among the models, MSE of 20.953, and RMSE of 4.577. This model seems to perform the best for predicting AnT with the highest R^2^ and the lowest MAE and RMSE.

**Fig 4:**
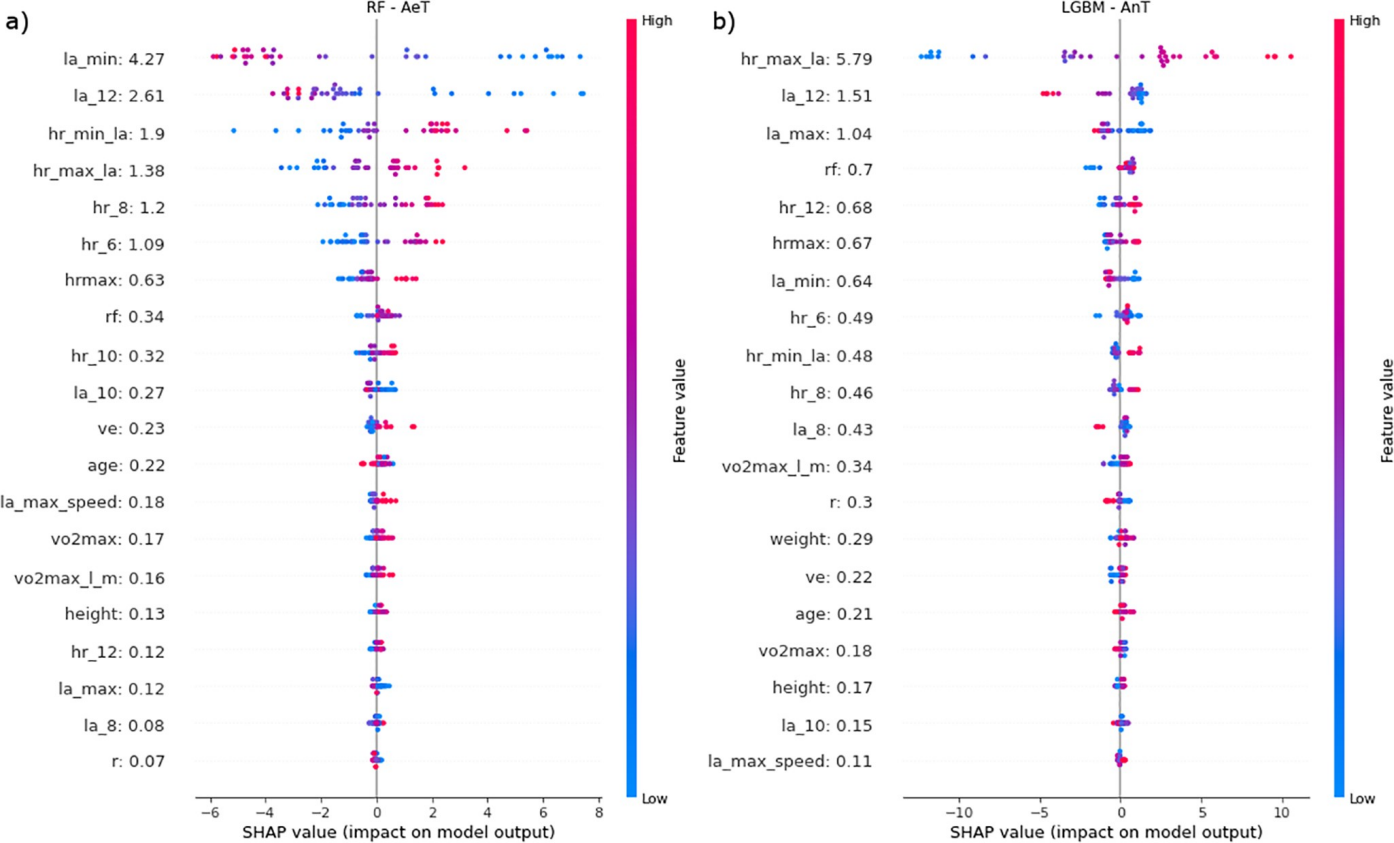
Feature importance analysis for the built prediction models that achieved the highest results: (a) Random Forest model for AeT prediction, (b) LGBM model for AnT prediction.

Using the SHAP tool, the importance of individual features was calculated for the ML models that obtained the highest prediction results (Fig 4). Determining the importance of features in predicting analyzed threshold levels can be helpful in optimizing athletic performance. It allows for tailored training programs that address specific physiological factors critical for improving endurance and anaerobic capacity. This analysis also supports personalized training plans, early detection of overtraining, and data-driven decision-making.

By analyzing the values obtained for the Random Forest model and AeT prediction, the following conclusions can be drawn (the obtained SHAP value is given in brackets after the feature name): the minimum lactic acid level (la_min: 4.27) is the most influential feature in predicting AeT for athletes. A higher minimum lactic acid level is strongly associated with a higher predicted AeT. This suggests that the ability to sustain exercise AnT a higher intensity without a significant increase in lactic acid production is a key factor in determining AeT in athletes. Lactic acid levels at load 12 km/h into exercise (la_12: 2.61) have significant importance in AeT prediction. A higher lactic acid level at this stage is a strong predictor of a higher AeT, indicating that an athlete’s ability to delay the accumulation of lactic acid is crucial for achieving a higher AeT.

A few features connected with HR measurement are strong predictors for AeT: the heart rate measured during the lowest level of lactic acid (hr_min_la: 1.9) and the heart rate measured during the maximum level of lactic acid (hr_max_la: 1.38) are other important features for predicting AeT. A higher minimum heart rate during lactate measurements is significantly associated with a higher predicted AeT, suggesting that cardiovascular fitness and heart rate response to exercise play a vital role in AeT. Similarly, a higher maximum heart rate during lactate measurements significantly correlates with a higher predicted AeT. Features related to HR measurement at the beginning of the performance test achieved significant SHAP values—respectively: HR at load 8 and 6 km/h (hr_8: 1.2, hr_6: 1.09) and maximum observed HR (hrmax 0.63). Other features from HR measurement group are less important: HR AnT load 10 and 12 km/h (hr_10: 0.32, hr_12 0.12) of the remaining features, respiratory frequency (rf: 0.34) had the highest level of influence. Conclusions regarding the built ML model for the prediction of anaerobic threshold grouped according to their impact on target values: high impact on AnT prediction (these features play a crucial role in determining an athlete’s AnT and are essential for optimizing training programs and enhancing athletic performance and endurance). The most influential feature in predicting AnT is the heart rate observed AnT maximum lactate acid level (hr_max_la: 5.79). A higher maximum heart rate strongly correlates with a higher predicted AnT, emphasizing the significance of cardiovascular fitness and the ability to reach and sustain a high heart rate during exercise. Lactic acid levels AnT load 12 km/h into exercise (la_12: 1.51) play a pivotal role in AnT prediction. Higher lactic acid levels at this stage are strongly associated with a higher predicted AnT, highlighting the importance of delaying the accumulation of lactic acid to achieve a higher AnT. Other vital features for AnT prediction are the observed maximum lactic acid level (la_max: 1.04) and the minimum lactic acid level (la_min: 0.64). Higher values of these features are positively related to a higher predicted AnT, emphasizing the ability of the athlete’s body to manage lactic acid levels during exercise. Heart rate observed at load 12 km/h (hr_12: 0.68) significantly influences AnT prediction. A higher heart rate at this point is linked to a higher predicted AnT, indicating the importance of cardiovascular response during exercise. Respiratory frequency (rf: 0.7) is another essential feature for AnT prediction. A higher respiratory frequency positively contributes to a higher predicted AnT, underscoring the significance of efficient breathing patterns during exercise. Moderate impact on prediction: the maximum heart rate observed during the performance test (hrmax: 0.67) moderately influences AnT prediction. Both heart rates at load 6 and 8 km/h of exercise (hr_6: 0.49, hr_8: 0.46) are moderate predictors of AnT, highlighting the importance of early heart rate response during exercise. A similar effect was observed for the heart rate measurement during minimum lactate acid level (hr_min_la: 0.48) and lactic acid levels at a speed of 8 km/h (la_8: 0.43). This group also includes body weight (weight: 0.29). Heavier athletes exhibit a moderate positive impact on AnT prediction, potentially due to muscle mass and power-to-weight ratios. Other features that have a moderate impact on AnT prediction are: respiratory rate (r: 0.3) and VO_2_max relative to lean body mass (vo2max_l_m: 0.34) is associated with a higher predicted AnT. The remaining features have a minor impact on AnT prediction for this model.

## Discussion

In sports science and athletes’ training planning, the parameters of physical capacity are useful. Among these, the lactate thresholds (aerobic and anaerobic) considered as key factors are widely used [1, 2]. However, the study showed that the aerobic threshold is generally more challenging to determine than the anaerobic threshold for several physiological and practical reasons.

Firstly, physiological complexity considers that AeT is the point at which lactic acid production in the muscles begins to exceed the body’s ability to remove it. It occurs gradually, making it challenging to pinpoint the exact moment when it happens. In contrast, AnT, which marks the shift from aerobic to anaerobic metabolism, typically manifests as a more distinct and observable change in physiological response, such as a lactate levels suddenly increasing in blood [1, 4]. Moreover, some difficulties may generate testing methods. Various methods can be employed to estimate AeT, including blood lactate measurements, gas exchange analysis, and heart rate monitoring [23–25]. These methods may yield slightly different results, adding to the complexity of AeT assessment. AnT, on the other hand, can be determined through a simpler approach, such as monitoring maximum heart rate, which tends to be more consistent. Additionally, the process possesses certain subjective experiences: athletes’ subjective experiences during exercise can affect their ability to identify AeT accurately. Some may perceive AeT as the point of increased discomfort or fatigue, which is subjective and not always an accurate indicator. AnT, typically marked by a pronounced decrease in performance, is easier to perceive.

Traditional methods for determining thresholds, such as blood lactate measurements and gas exchange analysis, are well-established and provide reliable data but can be time-consuming, invasive, and require specialized equipment. Machine learning models offer the advantage of handling complex, personalized data and identifying nonlinear relationships, potentially providing quicker and less invasive assessments. Identifying indicators and their quantitative values that limit the further development of the threshold-based technique is a great benefit for planning and adjusting training processes [40, 41].

However, these models rely heavily on the quality and quantity of the input data and may require extensive training datasets to achieve high accuracy. Trade-offs with the machine learning approach include the need for computational resources and the potential for overfitting, particularly with limited data.

Laboratory tests, which have so far been the best way to obtain reliable data, need to be conducted regularly to capture changes resulting from current training loads, diet, recovery, sleep quality, etc. The problem is gaining access to a sufficient amount of such data. Elite athletes, of course, conduct tests frequently enough, but access to their results is limited. Recreational athletes usually lack knowledge about the importance of these tests and often don’t have the time or financial resources for them. Current research proves, however, that recreational athletes also benefit significantly from CPET analysis by receiving advanced, personalized insights and guidance [41, 42].

One of the possible solutions to this problem may lie in the observed rapid increase in popularity of wearable devices over the past few years [43]. Sport watches and bands empower athletes of all levels to collect and analyze data, monitor performance, and make informed decisions to enhance their overall performance [44, 45].

Despite these challenges, machine learning models can offer significant improvements in efficiency and personalization, making them a valuable complement to traditional methods in both research and practical applications.

Our observations are consistent with the results presented in other studies [46], which also showed that assessing the aerobic threshold poses many difficulties.

**Fig 5:**
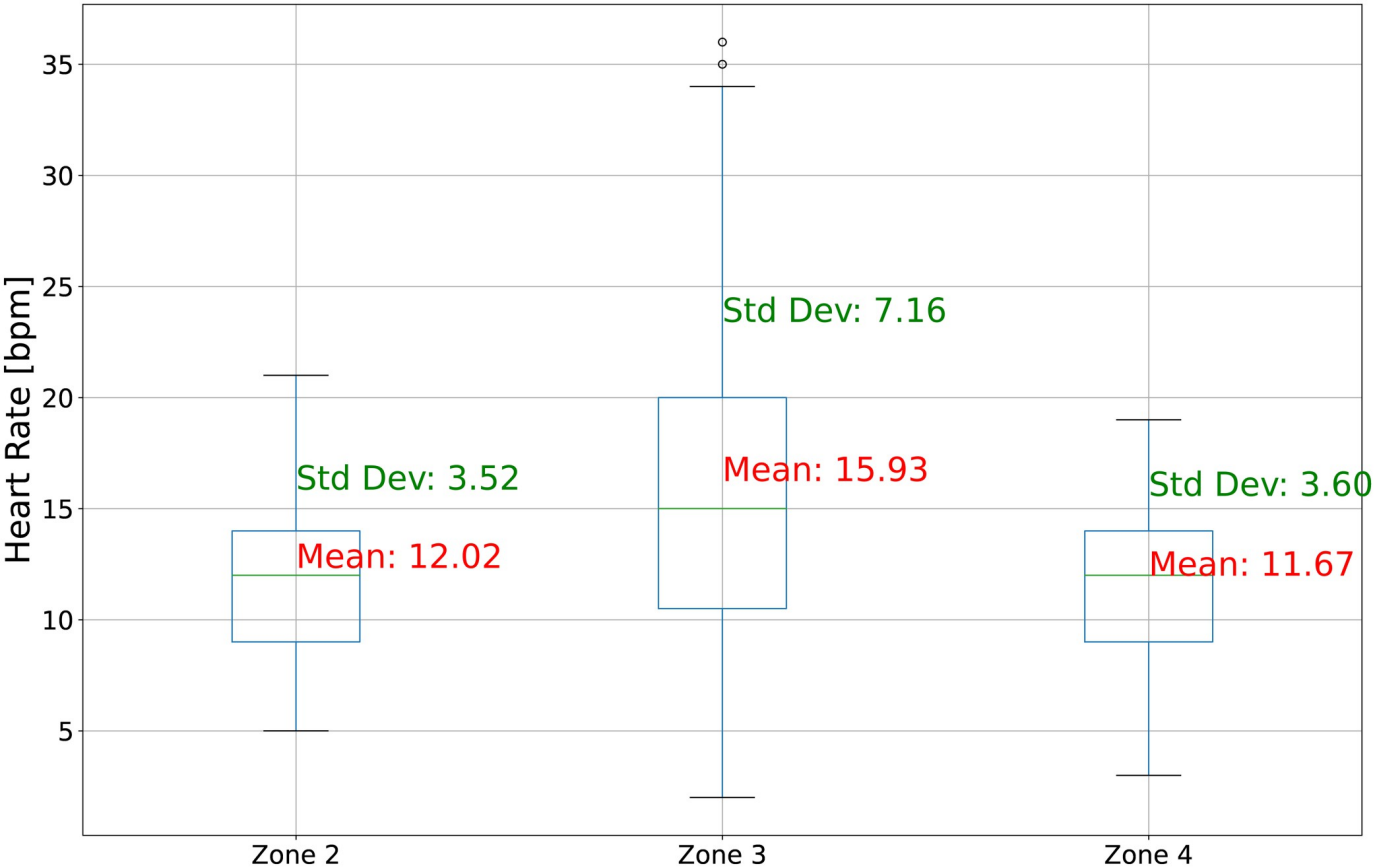
Descriptive statistics of the width of training zones resulting from the AeT and AnT.

Another critical consideration revolves around the overall precision of AeT and AnT determination and whether it is adequate for the purpose of planning training programs [1, 2]. Evaluating whether the level of accuracy achieved aligns with the requirements of effective training program design is essential. To analyze this issue, Fig 5 presents selected descriptive statistics of the width of training zones resulting from the AeT and AnT thresholds.

Analyzing Table 1, it can be observed that the Mean Absolute Error averaged 5 bpm for AeT and 3.5 bpm for AnT. This finding corresponds with the mentioned challenge in thresholds determination, which could be suitable for effective training proceedings. The aerobic zone (zone 2) is determined from the AeT threshold downwards, indicating that the boundary point is the HR level at the AeT threshold. Typically, Zone 2 has a range of 6 to 15 bpm. In this case, the average error in determining the threshold using ML will not significantly impact training if it is a deviation downward (below the AeT threshold), as it will fall within the expected working range, exerting the intended training stimulus on the athlete’s body. However, an upward deviation above the threshold indicates entry into the mixed zone. In this case, determining the zone in such a way would not be favourable for training. Shifting the zone above the threshold by 5 bpm is sometimes associated with a much higher level of lactic acid. In Zone 2, the primary focus is building aerobic endurance based only on the breakdown of fatty acids. Exceeding the threshold upward by 5 bpm activates glycogen breakdown, leading to the training of a different capacity—aerobic power. In turn, zone 4 is determined from the downward AnT threshold. Considering the level of lactic acid at the individual AnT threshold, where we observe deviations from around 3.2 mmol/l to about 6.8 mmol/l, in this case, an average error of 3.5 bpm for AnT determination using ML will not significantly impact the training process. Exceeding the AnT threshold by 3.5 bpm at the AnT threshold level will not significantly alter the lactic acid level.

The described method is subject to various limitations, such as precision of determination, psychological aspects of treadmill training, individual physiological factors or measurement errors. The psychological aspects of treadmill-based training and testing should not be overlooked. The need for artificial stimulation, such as running on a treadmill, can influence measurement results. Factors such as stress, running technique, and technical aspects of the treadmill (e.g., surface type, treadmill width) can impact the measurement outcomes. Additionally, measuring lactate levels during exercise introduces another layer of complexity.

The state of an athlete’s body is highly variable and influenced by multiple factors, including nutrition, hydration, sleep, training phase, and the time of day. These individual physiological factors can introduce variability and affect the accuracy of AeT and AnT determination. Measurement errors should be considered as well. The precision and accuracy of the measurement devices used in the assessment process may introduce specific measurement errors. Different devices operate with distinct levels of accuracy, resulting in specific sources of measurement error.

The current models were trained on data from a specific group of amateur athletes, which may not fully represent the broader athletic population, including professional athletes or those from different sports disciplines. Future research should focus on balancing the dataset [47] and validating these models across diverse athlete populations to ensure their broader applicability and reliability. Expanding the dataset to include athletes from various backgrounds, skill levels, and sports can significantly mitigate the problem of overfitting by providing the models with a more comprehensive range of training data. This will help understand how these models perform in various real-world scenarios and contribute to developing more robust and generalizable tools for athletic performance assessment. Additionally, incorporating data from different training environments and conditions will further enhance the models’ ability to adapt to the diverse situations athletes encounter, thereby improving the precision and effectiveness of performance predictions and recommendations. By continuously updating the dataset and retraining the models, researchers can ensure that the ML tools remain relevant and accurate over time, accommodating new trends and variations in athletic training and performance.

Understanding and addressing these limitations is vital for ensuring the reliability and applicability of the proposed AeT and AnT determination methods. One potential possibility for improving the effectiveness of determining AeT and AnT is incorporating a larger dataset. This can involve continuing the described performance tests for additional athletes, thereby increasing the sample size and allowing for more robust and generalizable conclusions. Another way for enhancement is to simultaneously measure heart rate variability (HRV) and/or concurrently measure heart rate variability during the assessments. HRV provides valuable insights into autonomic nervous system activity and can offer additional data for a more comprehensive understanding of an athlete’s physiological responses during exercise. Similarly, increasing the frequency of lactic acid measurement may potentially improve the accuracy of AeT and AnT determination via better monitoring of changes in lactic acid levels in real-time, which can provide more detailed data. The level of lactic acid may change dynamically in response to changing training intensity. More frequent measurements may allow capturing these changes and provide a more comprehensive picture of how your body responds. Moreover, determining the optimal time to measure lactic acid levels during training may be crucial. More frequent measurements may help identify moments of increased lactic acid production. These rationalizations may be impassable, e.g., there are many limitations that make such measurements very difficult. Nevertheless, each body responds individually to exercise, so more frequent measurements can help understand the unique response patterns to training in different people.

However, it’s worth noting again that the widespread adoption of personal wearable devices means that much of the ML process data is readily available. Automated threshold prediction, which considers basic physiological parameters like HR, HRV and respiratory rate, and information about current training status, recovery, diet, health, sleep patterns, etc., could offer a compelling alternative to regular expert-led laboratory tests [48].

It seems very likely that the rapidly growing database, which contains significantly more parameters than those considered in the traditional approach to determining AeT and AnT, could prove to be a breakthrough. Just as it is happening in other diagnostic applications, such as predicting diabetes mellitus or atherosclerotic cardiovascular risk prognostication [49, 50], ML will prove to be not only a more accessible solution but also more accurate. It will also allow us to notice and understand other previously unexplored physiological relationships, thereby contributing to better training management of elite and recreational athletes.

## Conclusion

The aerobic and anaerobic thresholds are critical physiological parameters impacting an athlete’s performance, training, and overall success in sports. Understanding these thresholds helps athletes and coaches make informed decisions about training, pacing, and competition strategies, ultimately contributing to improved athletic performance and well-being. ML is highly relevant in predicting AeT and AnT due to its ability to handle complex, personalized data, identify nonlinear relationships, and provide accurate predictions. It offers the potential for improved performance optimization, real-time monitoring, and innovative research in the field of sports science and athlete performance.

Based on the used evaluation metrics, the following models give the best predictions for:

- AeT: Random Forest RF has an R^2^ value of 0.645, MAE of 4.630, MSE of 44.450, RMSE of 6.667,
- AnT: LightGBM has a R^2^ of 0.803, the highest among the models, MAE of 3.439, the lowest among the models, MSE of 20.953, and RMSE of 4.577.

Determining the aerobic threshold proves more challenging than the anaerobic threshold due to both physiological intricacies and practical considerations. AeT represents a gradual transition, where lactic acid production surpasses removal capabilities, making its precise identification elusive. In contrast, AnT, signalling the shift from aerobic to anaerobic metabolism, typically brings about more distinguishable physiological changes, like a sudden surge in blood lactate levels. Additionally, the various testing methods and athletes’ subjective experiences further complicate AeT assessment. The simpler, more consistent methods used for AnT determination, such as monitoring maximum heart rate, add to the comparative complexity of AeT assessment.

Based on the outlined research experiment and a comprehensive review of existing literature in the field, the authors outline their intended research endeavors about the subject matter, which include:

- employing the newly devised methodology to assess the precision of heart rate zone determination by different training devices,
- examining how the physical condition of athletes impacts the precision of heart rate zone determination,
- exploring potential methods to enhance the precision of heart rate zone determination, such as incorporating diverse devices that monitor post-exercise bodily recovery states.

Continuing these studies with a significantly larger dataset could bring substantial benefits to the field of athletic performance analysis. A more extensive dataset would provide a richer and more diverse set of training examples, allowing the ML models to learn from a wider variety of physiological responses and performance metrics. This would improve the model’s ability to generalize across different types of athletes and training conditions, ultimately enhancing the robustness and accuracy of AeT and AnT predictions. Additionally, a larger dataset would enable the identification of subtle patterns and correlations that might be missed in smaller samples, leading to more refined and effective training recommendations.

Future research could explore integrating ML models into real-time athletic training devices. These models, trained on extensive datasets, could be embedded into wearable technology to provide athletes with immediate feedback on their AeT and AnT during training sessions. This real-time integration could enhance the precision of training adjustments, allowing athletes to optimize their performance more effectively. Additionally, future research could investigate the potential of these models to adapt dynamically as athletes’ physiological responses evolve, ensuring continuous improvement and adaptation of training regimens. Another promising avenue is these models’ potential to help prevent injuries. By continuously monitoring physiological indicators and recognizing patterns that precede injuries, ML models could provide early warnings and suggest modifications to training loads and techniques, thereby reducing the risk of injury and prolonging athletes’ careers.

The implications of this research extend beyond sports science. The ability of ML models to handle complex, personalized data and identify nonlinear relationships has potential applications in various fields, including healthcare, fitness, and personalized medicine. In healthcare, similar models could predict patient treatment responses or monitor chronic conditions in real-time. In the fitness industry, these models could personalize workout plans based on individual physiological responses, enhancing the effectiveness of fitness programs. Moreover, the techniques developed in this research could be applied to other forms of performance analysis, such as cognitive or workplace performance, where understanding and optimizing individual responses to stress and workload is crucial.

This research underscores the transformative potential of integrating advanced data analytics into personalized health and performance monitoring technologies. By incorporating these ML models into athletic training practices, real-time adjustments to training regimens can be made, optimizing performance and significantly reducing the risk of injury. This technology could revolutionize athletic training by providing precise, individualized feedback, thereby enhancing training efficiency and effectiveness. Additionally, continuous monitoring and dynamic adaptation of training loads can help prevent overtraining and burnout, leading to more sustainable athletic development and success.

## Supporting information

S1 File This is the S1 File dataset. This is the dataset used during the research.
(CSV)
